# A convolutional neural network with image and numerical data to improve farming of edible crickets as a source of food—A decision support system

**DOI:** 10.3389/frai.2024.1403593

**Published:** 2024-05-14

**Authors:** Henry Kyalo, Henri E. Z. Tonnang, James P. Egonyu, John Olukuru, Chrysantus M. Tanga, Kennedy Senagi

**Affiliations:** ^1^Data Management, Modelling and Geo-Information Unit, International Centre of Insect Physiology and Ecology, Nairobi, Kenya; ^2^@iLabAfrica, Strathmore University, Nairobi, Kenya; ^3^School of Agricultural, Earth, and Environmental Sciences, University of KwaZulu-Natal, Durban, South Africa

**Keywords:** insects, sound classification, transfer learning, machine learning, deep learning, decision support system

## Abstract

Crickets (*Gryllus bimaculatus*) produce sounds as a natural means to communicate and convey various behaviors and activities, including mating, feeding, aggression, distress, and more. These vocalizations are intricately linked to prevailing environmental conditions such as temperature and humidity. By accurately monitoring, identifying, and appropriately addressing these behaviors and activities, the farming and production of crickets can be enhanced. This research implemented a decision support system that leverages machine learning (ML) algorithms to decode and classify cricket songs, along with their associated key weather variables (temperature and humidity). Videos capturing cricket behavior and weather variables were recorded. From these videos, sound signals were extracted and classified such as calling, aggression, and courtship. Numerical and image features were extracted from the sound signals and combined with the weather variables. The extracted numerical features, i.e., Mel-Frequency Cepstral Coefficients (MFCC), Linear Frequency Cepstral Coefficients, and chroma, were used to train shallow (support vector machine, k-nearest neighbors, and random forest (RF)) ML algorithms. While image features, i.e., spectrograms, were used to train different state-of-the-art deep ML models, i,e., convolutional neural network architectures (ResNet152V2, VGG16, and EfficientNetB4). In the deep ML category, ResNet152V2 had the best accuracy of 99.42%. The RF algorithm had the best accuracy of 95.63% in the shallow ML category when trained with a combination of MFCC+chroma and after feature selection. In descending order of importance, the top 6 ranked features in the RF algorithm were, namely humidity, temperature, C#, mfcc11, mfcc10, and D. From the selected features, it is notable that temperature and humidity are necessary for growth and metabolic activities in insects. Moreover, the songs produced by certain cricket species naturally align to musical tones such as C# and D as ranked by the algorithm. Using this knowledge, a decision support system was built to guide farmers about the optimal temperature and humidity ranges and interpret the songs (calling, aggression, and courtship) in relation to weather variables. With this information, farmers can put in place suitable measures such as temperature regulation, humidity control, addressing aggressors, and other relevant interventions to minimize or eliminate losses and enhance cricket production.

## 1 Introduction

In recent years, edible insects have gained global attention as an under utilized source of food with great potential to contribute to future food and feed needs. It is also noted that insect farming has minimal damage to the earth due to low greenhouse gas emissions (Lange and Nakamura, 2021). Some of the edible insects which can be farmed for food are the crickets. The nutritional content of edible crickets, including vitamins, minerals, proteins, fats, essential amino acids, and flavonoids, is comparable or even superior to that of common animal protein sources like fish, poultry, and cow meat. Owing to their nutritional value and contribution to the livelihoods of many communities globally, over 2,000 species of insects are consumed by hundreds of millions of humans for millennia in more than 110 countries worldwide (van Huis, 2013; Kelemu et al., 2015; Verner et al., 2021). Unfortunately, most edible insects are currently harvested seasonally from the wild, and the harvests are declining due to the degradation of their breeding habitats. Farming of edible insects is in its infancy due to several reasons including limited/unavailable rearing protocols and poor adoption of technology among the farmers. Generally, the production of edible insects as a source of food and feed is low compared to the market needs (van Huis, 2013; Kelemu et al., 2015; Magara et al., 2021; Tanga et al., 2021; Verner et al., 2021).

Manual identification and understanding of insects is tedious, time-consuming, and subject to human error (Alonso et al., 2017; Potamitis et al., 2017; Kawakita and Ichikawa, 2019; Noda et al., 2019; Zhang et al., 2021). Automatic sound/audio signal processing can be improved using machine learning (Noda et al., 2016, 2019; Phung et al., 2017; Kawakita and Ichikawa, 2019). Machine learning has successfully been deployed in the identification and classification of insects based on their species (Phung et al., 2017; Zamanian and Pourghassem, 2017), acoustics (Amlathe, 2018; Kiskin et al., 2020; Zhang et al., 2021), wingbeats (Arpitha et al., 2021; Kim et al., 2021), etc. For instance, Kawakita and Ichikawa (2019) explored the classification of bees and hornets based on their flight sounds using the support vector machine (SVM) algorithm combined with Mel-frequency cepstral coefficient (MFCC) features. The model achieved significant recall and precision metric scores but faced challenges in classifying species with subtle differences in sound features. Zamanian and Pourghassem (2017) used multi-layered perceptron (MLP) and genetic algorithms to classify cicada species based on their sounds. Dong et al. (2018) employed convolutional neural networks (CNN) with enhanced spectrograms for insect recognition, while Tey et al. (2022) used spectrogram images and deep learning algorithms for cicada species recognition. These approaches achieved accuracy rates ranging from 77.78% to 99.13%. Kim et al. (2021) and Zhang et al. (2021) used CNN models with MFCC to classify insect sounds, achieving accuracy rates of 92.56% and 85.72% respectively.

Generally, crickets have certain characteristics/behaviors which when learned/known, can be key in informing the farmer what to leverage to improve the health of the insects to increase production. Such characteristics include the sound that informs the behavior/health of the crickets. For instance, crickets produce sounds to signify/mean certain behavior/activities (e.g., courtship, calling, aggression, etc.) (Alexander, 1961; Miyashita et al., 2016; Lin and Hedwig, 2021). The loud calling songs are meant to attract distant females, soft courtship songs initiate sexual behavior with nearby females, and aggression songs are produced when fighting for mates and territories. Keeping other factors (e.g., feeding rate, etc.) constant, these activities are mainly influenced by the temperature and humidity (Ulagaraj, 1976; Srygley, 2014; Niemelä et al., 2019). Therefore, using state-of-the-art machine learning algorithms, this study developed a novel insect sound synthesis decision support system to enable farmers to understand the health/status of their cricket farms and make meaningful decisions as they farm edible crickets as an alternative source of proteins and food. The novel approach can assist farmers improve the production of edible crickets as a sustainable source of food for humans compared to livestock farming and contribute to alleviating food insecurity and malnutrition challenges.

The following are the key contributions of the work reported in this paper:

- We studied the performance of shallow machine learning algorithms with numerical features and added weather variables features extracted from cricket songs. Moreover, the different numerical features were combined, trained on the shallow learning algorithms and their performances were evaluated.
- We investigated the important features of the extracted chroma frequencies and corresponding weather variables. Thereafter, the important chroma features were validated and mapped on the chromatic scale. The important weather variables were also discussed.
- We extracted image (i.e., spectrograms) features and trained several deep-learning CNN architectures. Weather variables were injected into the respective CNN architectures and merged with the image features in the deep learning architecture.
- We selected the best-performing machine learning model and deployed its pre-trained model on a decision support system (with a dashboard and notification system integrated) that can help farmers manage cricket farms.

This paper is organized as follows: Section 2 states the experimental setup that includes data collection, data preprocessing, feature extraction, and machine learning. Sections 3, 4 outline the key findings and their interpretation respectively. Lastly, Section 5 concludes the paper.

## 2 Materials and methods

### 2.1 Experiments setup and data collection

**Figure 1:**
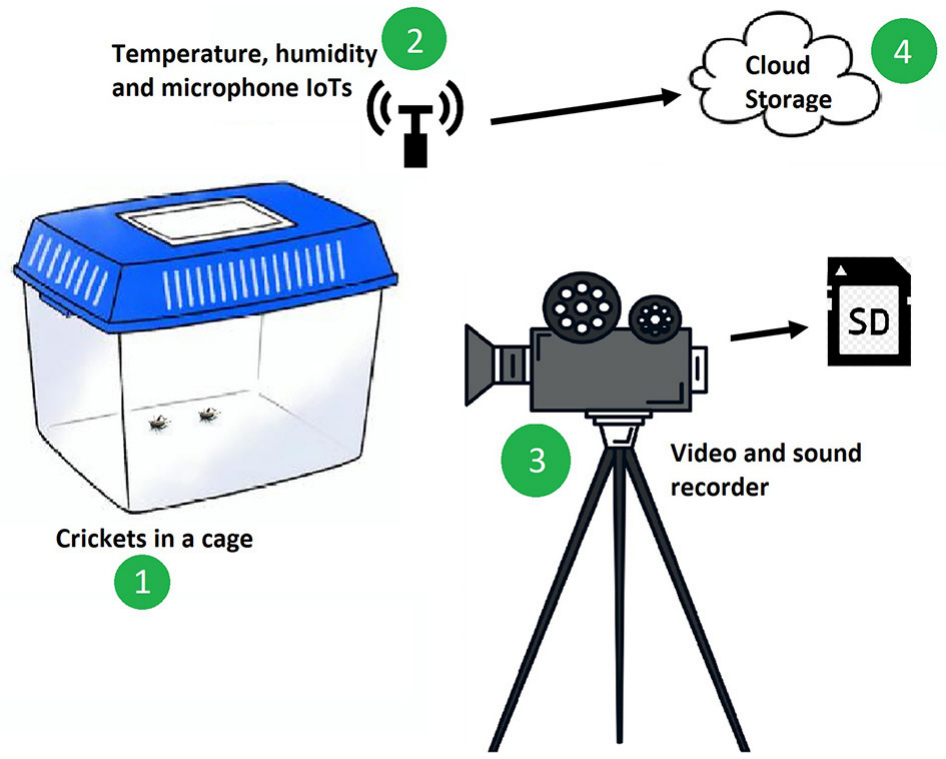
This illustration depicts the experimental setup used to study cricket (1) behavior and song production under varying temperature and relative humidity conditions. Temperature sensors or data loggers (2) were placed within the setup to continuously monitor and record temperature changes throughout the study, helping to understand the impact of temperature on cricket behavior and song production. Additionally, the video and sound recorder (3) continuously recorded audio and video data, allowing for observation and documentation of the crickets' songs and behavior throughout the experiment and the data was transferred to a hard disk. All data collected during the experiment were transferred to cloud for storage and analysis.

Video, sound, and associated weather data of the edible crickets, *Gryllus bimaculatus*, were collected in a laboratory (where environmental conditions were not controlled) as shown in Figure 1. The video was recorded using a Nikon Z6 II camera while temperature and humidity variables were recorded using an Internet of Things (IoTs) sensor installed in the laboratory. The temperature and humidity were transmitted to the International Centre of Insect Physiology and Ecology (*icipe*) virtual cloud at hourly intervals. During the data processing stage, the video footage was utilized to label the dataset, as it provided visual cues regarding the cricket behavior associated with the various songs they produce.

Cricket data at the nymph stage were not recorded since they do not chirp, i.e., their wings which enable crickets to produce sound are not fully developed. The female cricket's sounds were also not recorded as they do not produce any chirps (Jonsson et al., 2021; Lin and Hedwig, 2021). Therefore, this research processed sound signals in relation to the male crickets at the adult and mature stages. Data were collected for single males, male-male, or male-female to observe whether the crickets behave differently under different experimental setups; the specific days when the crickets were paired are shown in Table 1. The data was collected for over 24 hours for each pairing within the different stages for a period of 13 days continuously. The data collected consisted of 465 video recordings each 30 minutes long.

**Table 1:** A summary of data collection dates and the cricket pairing over the period of data collection.

**Date**	**Cricket pairing**
12th Jan 2023–14th Jan 2023	Male-Female
14th Jan 2023–15th Jan 2023	Male-Male
17th Jan 2023–18th Jan 2023	Male-Male
18th Jan 2023–20th Jan 2023	Male
24th Jan 2023–26th Jan 2023	Male-Female
26th Jan 2023–28th Jan 2023	Male-Male

Each cricket pairing was recorded for 2 days before a different cricket pairing was replicated.

### 2.2 Data preprocessing

**Figure 2:**
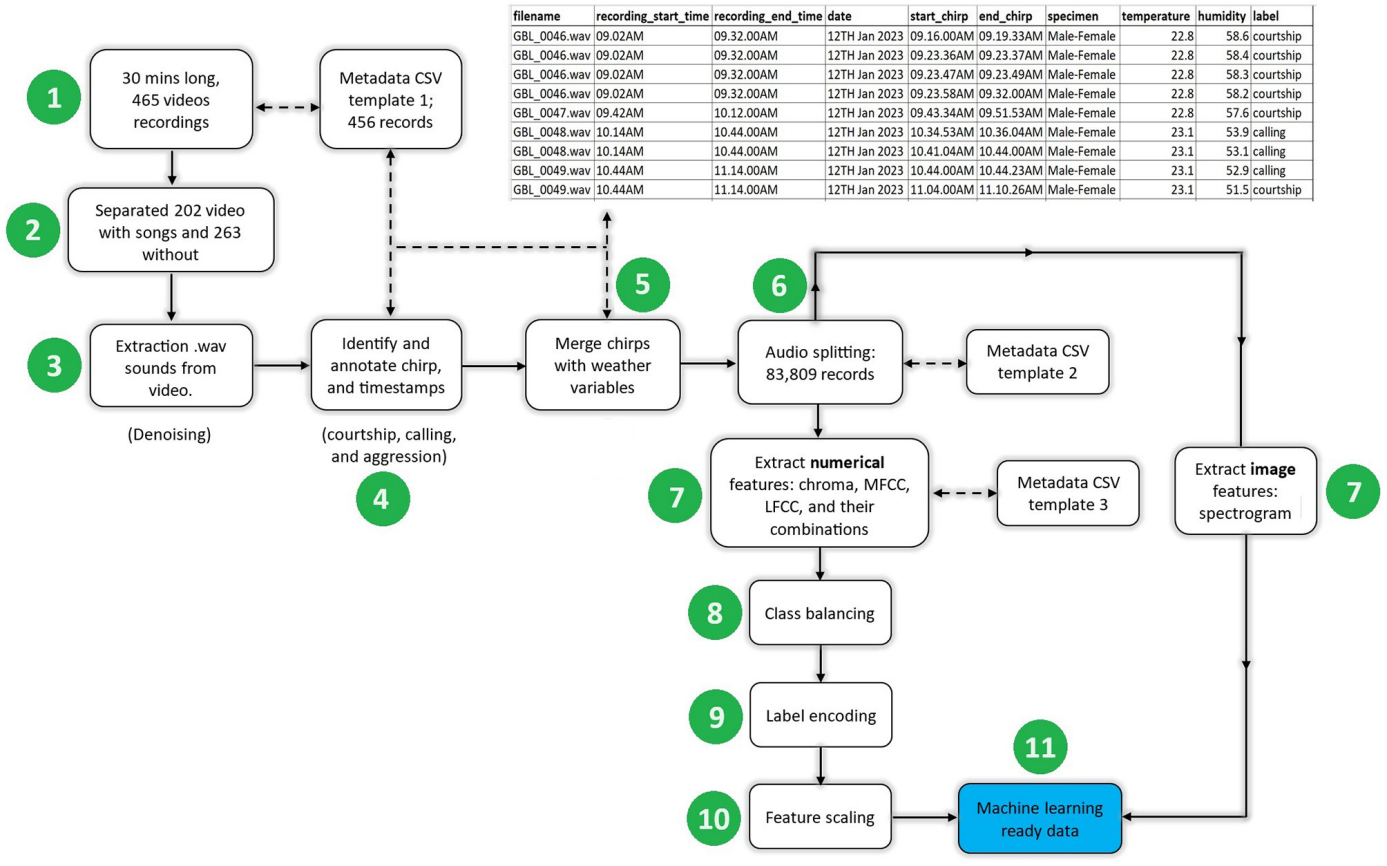
The pre-processing steps for the behavioral and weather data of the edible cricket *Gryllus bimaculatus* involved several stages, namely extracting cricket sound signals from the videos, removing noise from the extracted sound signals, annotating/labeling the sound signals, splitting sound signals, extracting features, class balancing, label encoding, and feature scaling.

Figure 2 illustrates the flow of the data cleaning process. At the end of the process, appropriate metadata were documented by filling a comma-separated values (CSV) template with the variables outlined in Table 2. The preprocessing steps are described below:

**Table 2:** A description of all the variables contained in the final metadata and used for exploratory data analysis.

**Variable**	**Description**
File name	File name of the recorded video
Recording start time	Time video recording was started
Recording end time	Time video recording was stopped
Date	Date the video was recorded
Start chirp	Time the cricket started to chirp
End chirp	Time the cricket stopped to chirp
Specimen	Whether the recorded cricket pairing was a single male, male-male, or male-female
Temperature	Room temperature in the laboratory
Humidity	Room humidity in the laboratory
Label	The actual behavior of the cricket as identified by a human expert

*Step 1: Recorded videos*. The 465 recorded videos were categorized into two groups: those containing cricket sounds and those without any cricket sounds.

*Step 2: Separating videos*. Information from 202 video recordings, each spanning 30 minutes and containing cricket sounds, was captured in a CSV metadata file. Following this, a human expert reviewed the videos, noting the timestamps of the start and end of each chirp. These timestamps were then appended to the CSV metadata file. A Python script (icipe, 2023a), integrating the MoviePy (Zulko, 2023) library, was used to extract sounds from the videos. The sounds were saved in the form of Waveform Audio File Format (i.e., .wav).

*Step 3: Denoising*. During the video and sound recording in the laboratory, various noises such as vehicle sounds, bird chirps, and human activity were inadvertently captured. To address this issue, Audacity (Audacity, 2023) software was used to denoise the .wav sound clips.

*Step 4: Extracting and annotating cricket chirps*. After the 30-min-long .wav files were extracted and denoised using a tailored Python script (icipe, 2023a), the timestamps recorded in Step 1 (recording the start and end of chirps) were employed to extract the cricket chirps/sounds. The denoised .wav files were subsequently saved in a separate folder and, the metadata associated with these files were documented in the CSV file (as shown in Figure 2, top right). The cricket species are known to produce three types of chirps/songs: aggression, courtship, or calling songs (Alexander, 1961; Miyashita et al., 2016; Lin and Hedwig, 2021). In this step, the extracted sound clips were annotated with the guidance of an entomologist, the domain expert. This procedure involved referencing the CSV metadata file, retrieving the exact start and end timestamps of cricket chirps, listening to the corresponding songs, observing cricket behavior in the video footage, and labeling the behavior (e.g., aggression, courtship, or calling) in alignment with the corresponding record in the metadata file. In data engineering, this process is commonly referred to as “labeling.”

*Step 5: Merging cricket chirps with weather variables*. The CSV metadata, described in the top right of Figure 2 was used to merge the annotated cricket songs and their corresponding temperature and humidity based on the chirps timestamp and the day of data recording.

*Step 6: Sound splitting (segmentation)*. In their natural behavior, cricket chirps can vary in duration, ranging from 0.4 seconds to 3 minutes (Jones, 1966; Mhatre and Balakrishnan, 2006). The duration for splitting sound signals has an impact on the size of the resultant feature matrix size. For example, a longer split duration leads to a larger feature matrix and consequently increases the training time of the algorithm (Gold et al., 2011). Conversely, splitting chirps into shorter lengths augments overall data volume, resulting in ample training data for models. To strike a balance, this study opted to divide cricket chirps into uniform 1-second segments, resulting in a total of 83,809 records.

*Step 7: Feature extraction*. Numerical and/or image features can be extracted from sound signals. The selected machine learning algorithm (s) dictates the type of features extracted. For instance, literature shows that shallow machine learning algorithms were trained on numerical features (Zhang and Guo, 2010; Yazgaç et al., 2016; Kawakita and Ichikawa, 2019; Noda et al., 2019), while deep learning algorithms were trained on numerical or spectrogram image features (Dong et al., 2018; Kiskin et al., 2020; Arpitha et al., 2021; Tey et al., 2022). Herein, we extracted numerical (chroma, MFCC, and Linear Frequency Cepstral Coefficients (LFCC)) and image (i.e., spectrograms) features since they were widely used by other researchers (Noda et al., 2016, 2019; Yazgaç et al., 2016; Zamanian and Pourghassem, 2017; Dong et al., 2018; Kawakita and Ichikawa, 2019; Tey et al., 2022).

Besides the independent (chroma, MFCC, and LFCC) features, this research combined chroma+MFCC, chroma+LFCC, and MFCC+LFCC and trained them on various shallow machine learning algorithms described in Section 2.3. The default hyperparameters were applied during feature extraction. The extracted features are described as follows:

*a) Mel-Scale Frequency Cepstral Coefficients* : MFCCs are coefficients that collectively make up a Mel-Frequency Cepstrum (MFC). An MFC is a representation of the short-term power spectrum of a sound signal based on a linear cosine transform of a log power spectrum on a nonlinear Mel scale of frequency (Le-Qing, 2011). This feature extracts a default of 13 numerical coefficients. MFCC is the commonly used feature for insects songs processing and has been used by (Zhang and Guo, 2010; Silva et al., 2013; Noda et al., 2016, 2019; Yazgaç et al., 2016; Phung et al., 2017; Amlathe, 2018; Kawakita and Ichikawa, 2019). This feature is more preferred because the frequency bands are equally spaced on the mel scale, which approximates the human auditory system's response more closely than the linearly-spaced frequency bands used in the normal spectrum. The feature was extracted using the *librosa.feature.mfcc()* function in the Python-based Librosa (McFee et al., 2023) library.

*b) Chroma:* Chroma features represent audio signals in 12 tonal variations (C, C#, D, D#, E, F, F#, G, G#, A, A#, and B). The 12 pitches indicate the amount of energy in each pitch class present in the signal. A pitch is separated into two components i.e., the tone height and chroma. The tone height represents the octave number and the chroma is a representation of the respective pitch spelling attribute. Octave represents 12 pitches. Conversion of audio to chroma can be performed using short-time Fourier transforms (STFT) or constant-q transform (CQT) (McFee et al., 2023). In this study, chroma STFT was used, which computes a chroma from a power spectrogram or waveform. The feature was extracted using the *librosa.feature.chroma_stft()* function in the Librosa (McFee et al., 2023) library. The extracted chroma features can be matched with different frequencies to determine the pitches within which the cricket songs fall.

*c) Linear-Frequency Cepstral Coefficient :* LFCC is a feature representation commonly used in audio signal processing and speech recognition tasks. LFCC has the same working as MFCC features and provides a linear-scale representation of the cepstral coefficients. This feature has been used in previous experiments by different authors (Potamitis et al., 2007; Silva et al., 2013; Noda et al., 2016, 2019; Yazgaç et al., 2016). The features were extracted using the Python-based *spafe* library. This research extracted the LFCC feature using the *spafe.features.lfcc.lfcc()* function.

*d) Spectrograms*: Spectrogram features are represented on a 2D image. The x-axis represents time of sequences of spectra, and color brightness on the other axis represents the frequency of the strength of each component at each time frame. Spectrograms show where there is high or low energy, and how energy levels vary over time (Ali et al., 2024). In insect song synthesis, spectrograms capture the temporal and spectral characteristics of the insect sounds. The features were extracted using the *librosa. feature.melspectrogram ()* function in the Librosa library.

*Step 8: Class balancing*. In machine learning modeling training an algorithm with imbalanced data leads to the model learning too much of the majority features than the minority. The dataset was explored to check for class imbalance (i.e., balanced labels/classes) based on the target feature. To handle the class imbalance problem, the Synthetic Minority Oversampling Technique (SMOTE) was used. SMOTE creates a new dataset and oversamples by introducing some variance in the minority class. It works by finding the nearest neighbors of the minority class and drawing a vector of each of those points. As such, the method increases the number of minority class instances (to a level set by the user) in the neighborhood, thereby assisting the classifiers in which the data will be fitted to improve their generalization capacity (Fernández et al., 2018).

*Step 9: Label encoding*. Machine learning algorithms typically work with numerical data and therefore converting categorical labels into numerical labels enables the algorithms to capture the ordinal or nominal relationship between categories. In this context, the labels (calling, aggression, and courtship) were encoded using *preprocessing.LabelEncoder()* function of Sklearn (Pedregosa et al., 2011) Python library. This ensured that the predictor variables could be correlated with the target variable for easier classification.

*Step 10: Feature scaling*. This technique is used to normalize independent variables within a certain range. Feature scaling ensures that all features contribute proportionally to the distance calculations and features with larger scales cannot dominate the distances, leading to biased results (Disha and Waheed, 2022). In this study, the data (MFCC, LFCC, spectrograms, and weather numerical variable) were normalized using the min-max (Pedregosa et al., 2011) scaler defined in Equation 1 where *X* is the original value, *X*_*max*_ is the maximum value and *X*_*min*_ is the minimum value. The scaler normalizes data within a range of 0 to 1. This study notes that chroma features were already within a scale of 0 and 1, as such, not normalized.


(1) $$\begin{array}{l} X_{norm}=\frac{X-X_{min}}{X_{max}-X_{min}} \end{array}$$

*Step 11: Machine learning ready data*. After the data normalization, the data was machine-learning-ready. The following section explains machine learning modeling steps.

### 2.3 Machine learning modeling

**Figure 3:**
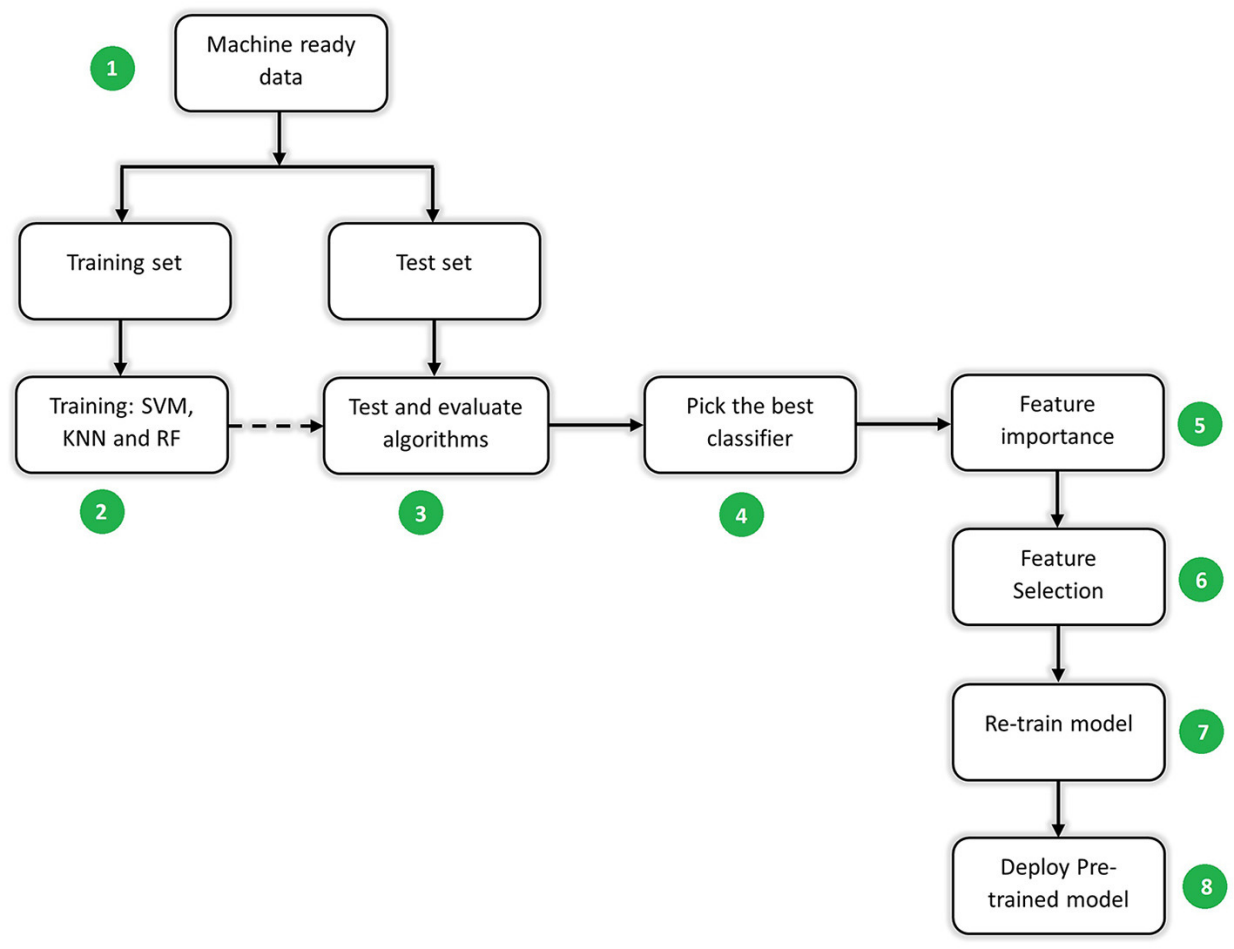
A summary of machine learning steps from splitting machine learning ready data (1), training various classification algorithms (2), testing and evaluating the model performance (3), picking the best classifier algorithm evaluated on test data (4), ranking features in order of their importance based on the best classifier (5), selecting the number of features which best predict the cricket songs (6), re-training the model with the selected features (7), and deploying the pre-trained model (8) as a .h5 file.

Figure 3 shows the steps in machine learning modeling. They are described in the steps below.

*Step 1: Data splitting*. The data (83,809 records) were split into train and test sets in a ratio of 70:30 respectively using the *sklearn.model_selection.train_test_split* function in the Sklearn (Pedregosa et al., 2011) Python library. By dividing the available data into separate training and testing sets, the model was trained on a portion of the data and its performance was evaluated on the unseen data. This helps in estimating how well the model generalizes to new/unseen instances. Data splitting helps in preventing data leakage, which occurs when information from the test set inadvertently influences the model training. Keeping the test set separate ensures that the model is evaluated on unseen data, providing a more accurate assessment of its performance (Joseph, 2022; Joseph and Vakayil, 2022).

**Figure 4:**
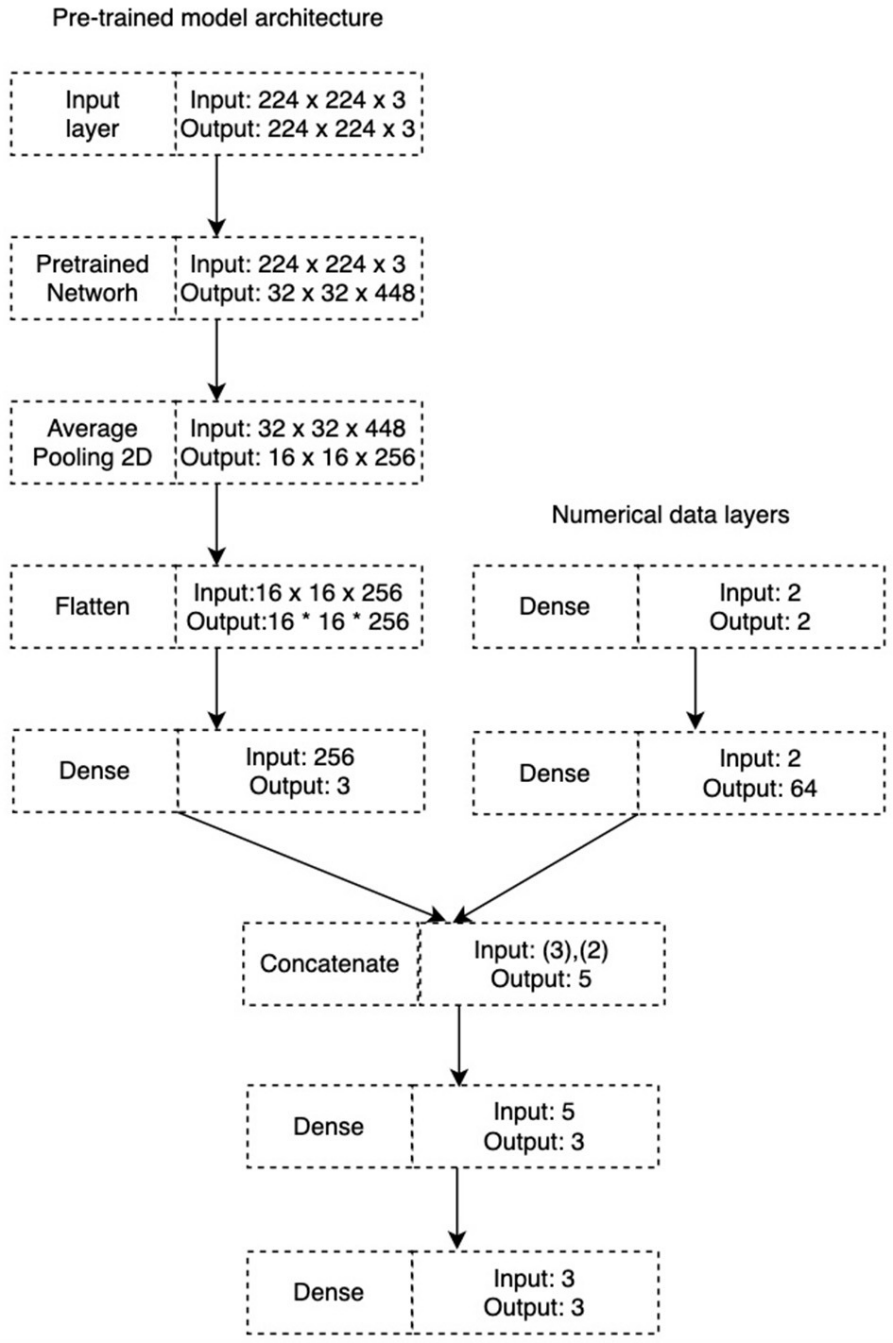
The general architecture of the ResNet152V2, EfficientNetB4, and VGG16 pre-trained models. The numerical data (i.e., temperature and humidity) layer was injected and merged in the pre-trained deep learning model.

*Step 2: Training machine learning algorithms*. This study implemented both shallow and CNN machine learning algorithms. These algorithms are imported from the Sklearn (Pedregosa et al., 2011) Python library and executed. The single features (described in Section 2.2, Step 7) and a combination of them were trained on SVM, k-nearest neighbors (KNN), and RF shallow machine learning algorithms which were configured with their default hyperparameters. Whereas spectrogram image features (described in Section 2.2, Step 7) were trained on CNN deep learning algorithms. These algorithms are briefly discussed as follows:

a) Support Vector Machine : This algorithm is used for both regression and classification tasks. In the algorithm, each data item is plotted as a point in n-dimensional space. The n-dimensional space represents the number of features to be classified in the model. Classification is done by finding an optimal hyperplane that separates the n-classes (Suthaharan and Suthaharan, 2016).
b) K-Nearest Neighbor : The algorithm evaluates the similarity between the new data and available cases and puts the new case into the category that is most similar to the available categories. The algorithm stores all the available data and classifies a new data point based on the similarity. When new data appears, it is easily classified into a well suit category, using the KNN algorithm (Kramer and Kramer, 2013).
c) Random Forest : This algorithm is mainly used for classification and regression problems. The algorithm consists of *N* decision trees trained on bootstrap random subsets of the data. RF utilizes ensemble learning, a technique that combines many classifiers to provide solutions to complex problems. The algorithm makes use of feature bagging, which has the advantage of significantly decreasing the correlation between each decision tree and thus increasing its predictive accuracy on average (Breiman, 2001).
d) Convolutional Neural Network : CNN is a deep neural network designed to process grid-like data such as images, audio spectrograms, and time-series data. The algorithm works by assigning weights and biases to the input features based on the importance of various objects in the input image. CNN processes data in a 1D array and 2D array. Different architectures (i.e., EfficientNetB4, ResNet152V2, and VGG16) of CNN were used in this study. These algorithms are pre-trained models built on the ImageNet dataset using the Keras framework. The initial layers of the pre-trained models were frozen, and the other layers of the models were fine-tuned on the acoustic dataset. The three models had similar architecture consisting of the average pooling 2D, the flattened layer, and the dense layer consisting of Relu activation and 256 neurons. This was followed by a final dense layer consisting of softmax activation and 3 neurons (aggression, calling, and courtship) which were the class labels to be predicted. For the loss, categorical_crossentropy was used since we only wanted to predict one class at a time. To prevent overfitting of the model, the early stopping methods with the patience of 10 and 50 epochs each with 58 steps were implemented. Table 3 contains optimization settings that were used across the pre-trained models. The pre-trained models took images (i.e., spectrogram features) as inputs. Numerical data (i.e., temperature, and humidity) were important variables in understanding the songs produced by the crickets and equally affected their growth. Therefore, the numerical data was injected and merged in the deep learning model architectures as shown in Figure 4.

**Table 3:** Hyperparameter settings used with the pre-trained models.

**Hyperparameter**	**Value**
Resolution	224 × 224
Color channels	3
Batch size	32
Transfer learning	Based on Imagenet
Validation size	0.3
Optimizer	Adam
Learning rate	0.001
Early stopping	True
Max epochs	50
Patience	10 based on val loss

*Step 3: Testing and evaluating the machine learning algorithms*. There are different evaluation metrics for classification problems such as accuracy, F1-score, confusion matrix, precision, and recall (Vujović et al., 2021). The choice of an evaluation metric depends on the problem one is investigating and the type of data one is dealing with. This study employed the F1-score and accuracy to evaluate the performance of the machine learning algorithms. The F1 score was selected due to the context of the problem (multi-class classification) and the nature of the data; which had imbalanced classes. It is noted that accuracy tends to underestimate classes with a smaller number of samples in relation to those with a larger number (Steiniger et al., 2020). Therefore, the accuracy score was chosen to compare its performance against the F1-score as it was the most used evaluation metric across many insects' song classification problems (Silva et al., 2013; Noda et al., 2016, 2019; Amlathe, 2018; Kim et al., 2021). Accuracy and F1-score performance metrics were used to evaluate the SVM, KNN, RF, and CNN algorithms discussed in the previous section. Based on their performance, the best classifier was selected.

The F1-score and accuracy performance evaluation metrics are discussed below:

a) Accuracy: It measures the number of correct predictions expressed as a percentage of the total number of predictions. A lower value of accuracy means the classifier predicts the wrong label, while a value closer to 100 means the classifier correctly classifies the labels. Accuracy can be calculated from the confusion matrix, which is a tabular representation of the performance of the classification model. The confusion matrix shows the number of true positives (TP), which represent the instances that are correctly classified positively as the target class. True negative (TN) is the number of instances correctly classified as the negative class. False positive (FP) is the number of instances incorrectly classified as positive. False negative (FN) is the number of instances incorrectly classified as the negative class. The values of (TP, TN, FP, and FN) are plugged in Equation 3 to calculate accuracy (Jeni et al., 2013; Han et al., 2022). Accuracy was calculated using the *accuracy_score* function in the Sklearn (Pedregosa et al., 2011) Python library.
b) F1-score: This metric measure combines both precision and recall, achieving a balance of both. Precision measures the proportion of true positive predictions out of all positive predictions made by the model. Recall measures the proportion of true positive predictions out of all actual positive instances in the dataset. F1-score is common in evaluating the performance of models built from imbalanced datasets, as it is not influenced by the majority class. There are 3 average techniques used with F1-score: macro, micro, and weighted. The macro technique gives equal weights to all classes making it suitable for balanced datasets, while the micro technique works by dividing the sum of the diagonal cells of the matrix by the sum of all cells. The weighted technique accounts for class imbalance by computing the average of binary metrics weighted by the number of samples of each class in the target (Pedregosa et al., 2011). F1-score was calculated using Equation 2 (Han et al., 2022). F1-score was calculated using the *f1_score* function in the Sklearn (Pedregosa et al., 2011) Python library

(2) $$\begin{array}{l} F1-score=\frac{2*Precision*Recall}{Precision+Recall} \end{array}$$

(3) $$\begin{array}{l} Accuracy=\frac{TP+TN}{TP+TN+FP+FN} \end{array}$$

*Step 4: Picking the best classifier*. The algorithm in the shallow and deep learning categories that gave the best performance metrics was selected.

*Step 5: Feature importance, feature selection, and re-training*. Feature importance makes us understand which features have the most influence on the model's prediction (Casalicchio et al., 2019). With deep learning, the model automatically locates important features (Liu et al., 2021), and therefore determining feature importance is not easy. Nonetheless, some shallow machine learning algorithms can perform feature importance, such as random forest, and support vector machine. Therefore, in this study, the best-performing shallow machine learning algorithm was selected to perform feature importance and feature selection based on the manually extracted features that were used to train the algorithm. Feature importance was carried out using the inbuilt *feature_importances_* function in Sklearn (Pedregosa et al., 2011) Python library. The function derives the list of important features using Gini impurity (Disha and Waheed, 2022), defined in Equation 4, where *P*_*p*_ refers to the fraction of positive samples and *P*_*n*_ refers to the fraction of negative samples of the total number of samples.


(4) $$\begin{array}{l} Gini\;Impurity=1-P_{p}^{2}-P_{n}^{2} \end{array}$$

Feature selection involves selecting the model inputs that best inform the model's prediction. Selecting the most relevant features helps improve model performance by reducing overfitting. Irrelevant or noisy features introduce unnecessary complexity to the model, leading to a poor generalization of unseen data. Therefore, feature selection enables the model to focus on the most informative features, resulting in better performance, lower variance, and improved predictive performance. It also reduces the computational cost of training and making predictions because of the fewer features. This study employed feature selection to identify a set of features out of those that were ranked (using important features). The algorithm was retrained with the selected features.

### 2.4 Deployment: the decision support system

For the farmer to interact with the system, and get insights (i.e., aggression, courtship, or calling) that inform the health status and activities of the insects under production, it was necessary to build and deploy the intelligent system (with the best shallow/deep machine learning pre-trained model at the back-end) on the web for universal access. The web application was developed on Django (Django, 2023) and deployed on a virtual machine instance in *icipe's* virtual private network on the cloud.

## 3 Results

### 3.1 Class labels

The three call types (i.e., aggression, calling, and courtship) of the *Gryllus bimaculatus* cricket species were observed (based on the video recordings noted in Section 2.2, Step 1).

### 3.2 Behavior of crickets and time

**Figure 5:**
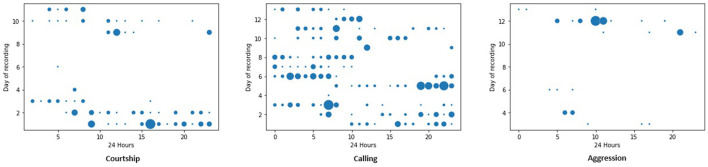
Different behavior recorded over 24 h in 13 days. A courtship song is produced by male crickets toward female crickets to initiate mating. A calling song is produced by male crickets to attract the attention of female crickets. Aggression song is produced by male crickets to male crickets in the fight for territory or females.

Figure 5 shows the singing behavior of crickets observed in 24 hours over the period of data collection. Calling songs were distributed across the hours of the day and on all the days. Generally, courtship and aggression songs were observed to occur on specific days.

### 3.3 Effect of temperature and humidity on cricket songs

**Figure 6:**
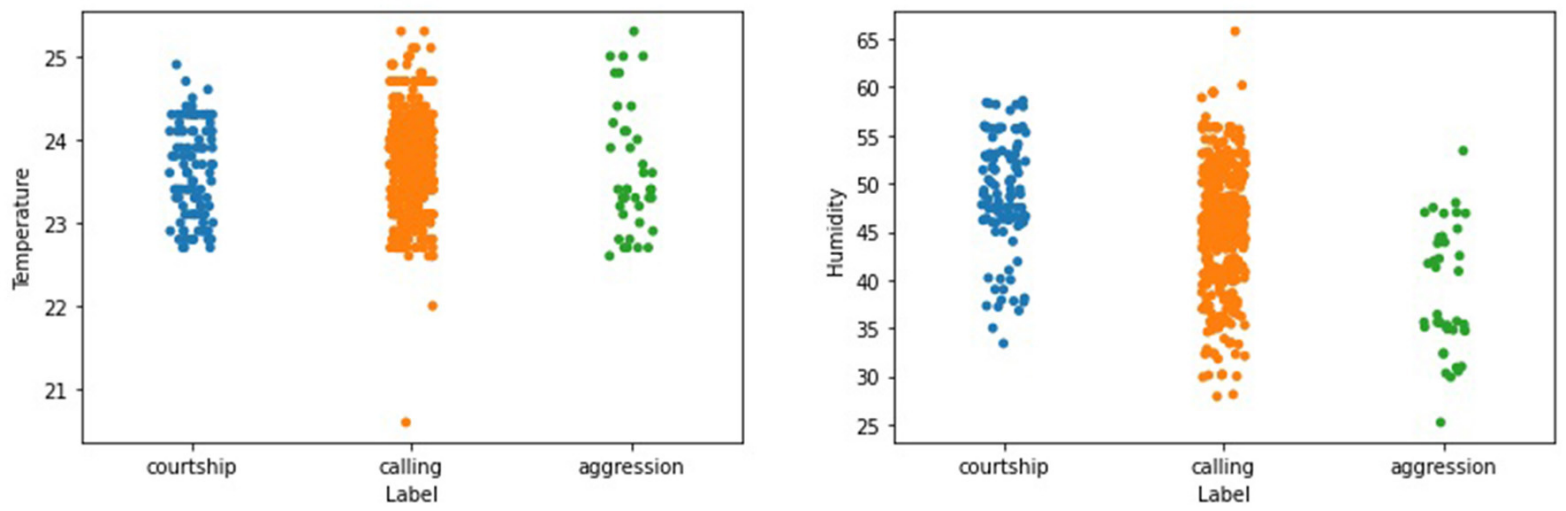
An illustration of how the cricket songs vary with different temperature and humidity values recorded.

The lowest and highest temperatures were 20.2 and 25.5°C while the lowest and highest humidity recorded was 28.3 and 59.4% RH respectively throughout data collection under natural conditions. Figure 6, shows how the different cricket songs change under the recorded temperature conditions. It is observed that the songs were recorded at temperatures above 22.5°C to the maximum recorded temperatures, i.e., 25.5°C (as shown in Table 3); a range of approximately 2°C. Moreover, Figure 6, shows that most courtship and calling songs were recorded between a humidity of 35 and 55% RH. Most aggression songs were recorded between 30 and 55% RH and were generally evenly distributed. Generally, songs concentrated on the range of approximately 45% RH.

### 3.4 Training and evaluating machine learning algorithms

The results of training RF, SVM, and KNN with both single and combined features as well as CNN architectures with spectrogram features are reported below:

#### 3.4.1 Single features in shallow learning algorithms

Table 4 summarizes the performance (accuracy and F1-score) results of the features trained on SVM, KNN, and RF machine learning algorithms trained with single features and fused with temperature and humidity variables. The best classifier was RF with an accuracy of 0.9277 and F1-score of 0.9394 when trained on chroma features.

**Table 4:** A summary of the performance of the single and combined features trained on the various algorithms and evaluated on accuracy and F1-score.

**Feature**	**Classifier**	**Accuracy**	**F1-score**
**Single features**			
Chroma	SVM	0.7692	0.8233
	KNN	0.8739	0.8928
	RF	0.9277	0.9394
MFCC	SVM	0.7118	0.7990
	KNN	0.8651	0.8885
	RF	0.9299	0.9264
LFCC	SVM	0.8554	0.7887
	KNN	0.7600	0.9275
	RF	0.8084	0.9301
**Combined features**			
Chroma+MFCC	SVM	0.7706	0.8246
	KNN	0.8816	0.8974
	RF	0.9441	0.9463
Chroma+LFCC	SVM	0.7699	0.8232
	KNN	0.8077	0.8480
	RF	0.9240	0.9288
MFCC+LFCC	SVM	0.5541	0.6810
	KNN	0.7971	0.8423
	RF	0.9294	0.9271

#### 3.4.2 Combined features in shallow learning algorithms

The merged chroma+MFCC (25 features), chroma+LFCC (25 features), and MFCC+LFCC (26 features) and fused with temperature and humidity variables were trained on SVM, KNN, and RF machine learning algorithms and the results are recorded in Table 4. The best classifier was RF when trained on chroma+MFCC with accuracy and F1-score of 0.9441 and 0.9463 respectively.

#### 3.4.3 Spectrograms in deep learning algorithms

The extracted spectrograms and the weather data were injected and concatenated into the pre-trained models, and the results were recorded in Table 5. The best pre-trained model was ResNet152V2, with accuracy and F1-score of 0.9942 and 0.9854 respectively.

**Table 5:** A summary of the performance of the CNN architectures.

**Features**	**Architecture**	**Accuracy**	**F1-score**
Spectrogram + humidity + temperature	ResNet152V2	0.9942	0.9854
Spectrogram + humidity + temperature	VGG16	0.9721	0.9721
Spectrogram + humidity + temperature	EfficientNetB4	0.9315	0.9322

### 3.5 Selecting the best classifier, feature importance and feature selection

**Figure 7:**
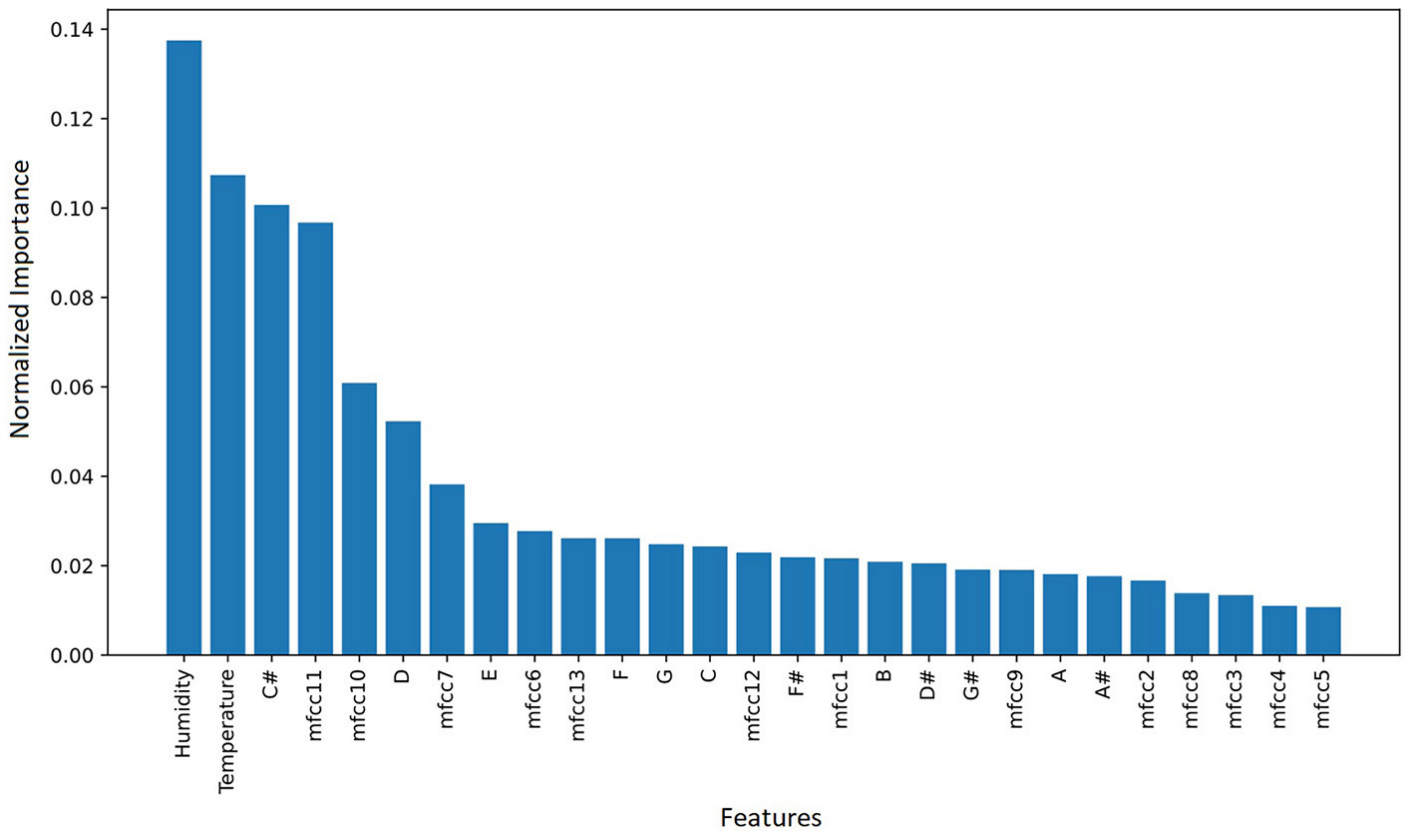
A graphical summary of the variable's contribution to the performance of the RF classifier in descending order. The top 6 variables were humidity, temperature, C#, mfcc11, mfcc10, and D variables.

**Figure 8:**
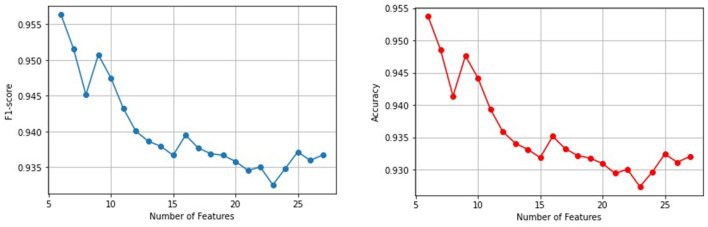
A graphical representation of RF classifier performance from 6 variables to 28 variables increased incrementally, and evaluated with accuracy and F1-score.

The results of training SVM, KNN, RF, and pre-trained models with single and combined features and spectrograms that were fused with weather variables (temperature and humidity) are documented in Sections 3.4.1, 3.4.2, and 3.4.3. From the results, deep learning pre-trained models have a better performance than shallow machine learning models. ResNet152V2 was the best model among all the models trained, with a classification F1-score and accuracy of 0.9854 and 0.9942 respectively. With shallow machine learning algorithms, the RF algorithm was the best classifier when trained with chroma+MFCC, temperature, and humidity features. Figure 7 illustrates the features in their order of importance obtained from RF as the best classifier. In that order, starting from the 6^*th*^ feature, the other variables were added incrementally, trained with the RF algorithm, and evaluated on F1-score, and accuracy results are summarized in Figure 8. It was observed that the top 6 features (humidity, temperature, C#, mfcc11, mfcc10, and D) had the best F1-score and accuracy of 95.63% and 95.37% respectively, which is still outperformed by the deep learning pre-trained models.

### 3.6 Deployment: the decision support system architecture

**Figure 9:**
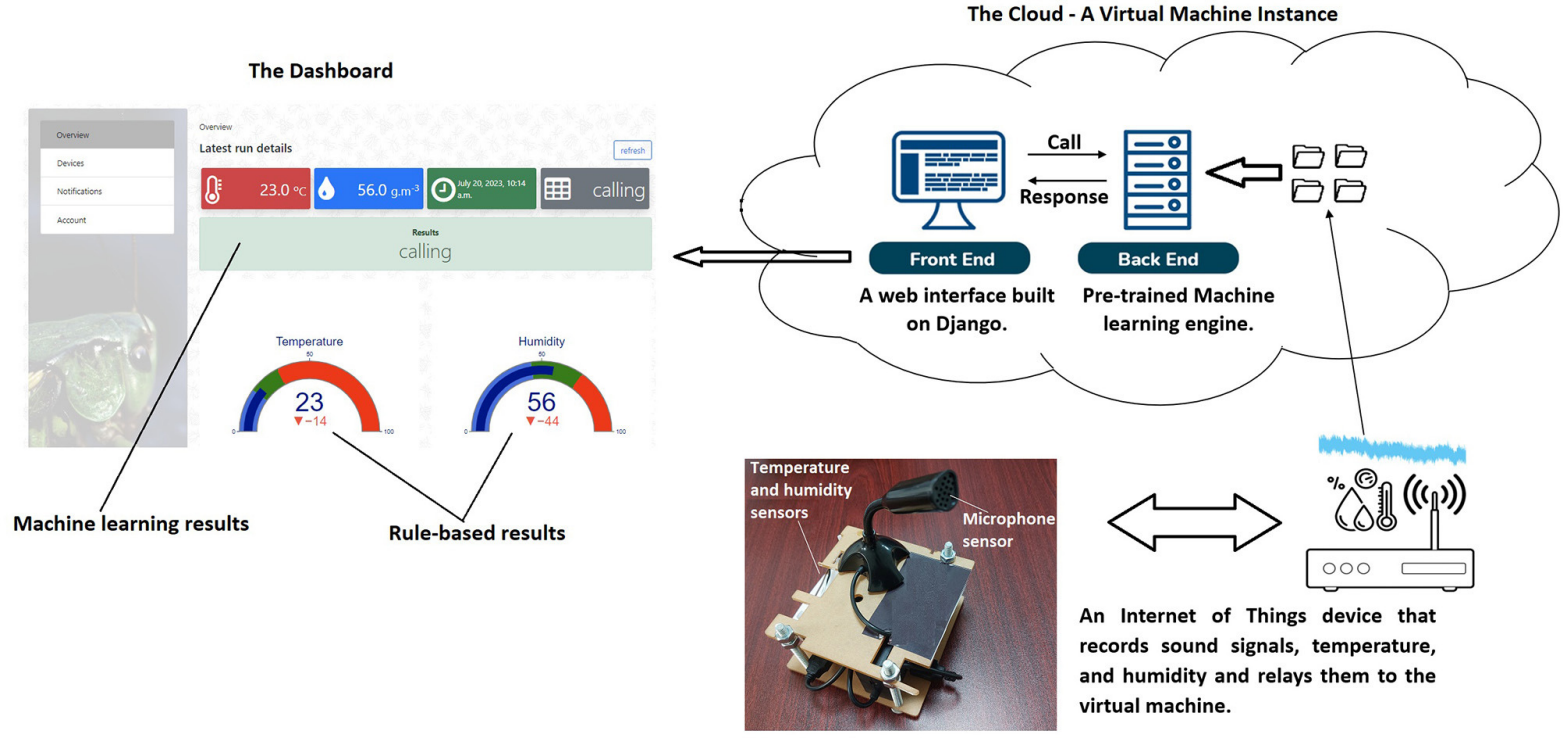
Deployment of the decision support system. After every 5 min, the IoT device captures and transmits 3-second .wav files, temperature, and humidity data to the virtual private network through a built-in WIFI module. Thereafter, after every 5 min, the system's back end fetches the latest records (.wav files, temperature, and humidity), preprocess them, and passes that to the pre-trained machine learning engine for prediction and the results are rendered on the front end dashboard.

Figure 9 gives the overall architecture of the developed decision support system that was deployed (accessible here icipe, 2023b) on a Kubernetes (Kubernetes, 2023) orchestration system. Generally, the system had three main components: data collection done by the IoTs, the back end that processes the collected data, and the front end for visualizing the results. These components are discussed in detail in Section 4.6.

## 4 Discussion

### 4.1 Class labels

The class labels (calling, courtship, and aggression) of cricket songs were similar to categories of cricket songs reported by other researchers (Alexander, 1961; Miyashita et al., 2016; Lin and Hedwig, 2021). Based on the distribution of those classes, Sections 2.2 (Step 8) and 3.1 identified and treated (using SMOTE) the class imbalance problem to prevent engineering a biased machine learning model that could understand and interpret the majority class more than the minority one. After resolving the class imbalance problem, we anticipate an equal/unbiased interpretation of the classes leading to a better performance of the model (Wang et al., 2016; Deng et al., 2022). Besides recording more calling songs compared to courtship and aggression songs, the study by Doherty (1985) also recorded more calling songs of the *Gryllus bimaculatus de Geer* cricket species and noted that the calling songs are more important than others since they trigger recognition and elicit phonotaxis (movement toward males) in female crickets. This could be a natural behavior for the survival (males calling the females to mate) of the crickets. Generally, the courtship songs are linked to mating. The cricket aggression songs are linked to the fighting behavior of the crickets. The calling songs are linked to oviposition, i.e., the males calling and the females laying eggs. Calling songs attracted the females toward the males for mating.

### 4.2 Behavior of crickets and time

As noted in the previous section, crickets call to attract possible mates. A calling song is produced in all instances of cricket pairing highlighted in Table 1. In Section 3.2, it was stated that the calling songs were produced throughout 24 hours. Table 1 indicates the days the crickets were paired in the rearing area, comparing that with the calling songs, this study notes that in all pairs, the males produced calling songs throughout the day. This was important to attract the females for courtship, mating, and reproduction.

In Section 3.2 it was evident that the courtship song was produced only when the male was paired with the female cricket. We observe that this song was produced on days 0, 1, 2, 3, 8, 9, and 10 when the male was paired with the female as observed in Table 1; since the song produced by males was to initiate the mating process with the females. In the same section, we observed that the aggression song was produced on days 3, 4, 11, and 12 when the male was paired with the male as observed in Table 1. This confirms that the aggression song was produced by males toward males in-fight for dominance or territory.

### 4.3 Effects of temperature and humidity on cricket songs

As stated in Section 3.3, the distribution of courtship, calling, and aggression songs were within a temperature range of 22.5 and 24.5°C. This study shows that temperature and humidity were quite influential on the type of songs produced by the crickets which is supported by the findings of Cheney et al. (2018) who found out that moderate/high temperature and humidity increased crickets' chirping rate. This also coincides with the findings of Doherty (1985) who found that crickets produced more calling songs within a temperature range of 15 and 35°C. Moreover, Niemelä et al. (2019) found that cricket's behavior was temperature-dependent since an increase in temperature increased their ability to express their behavior and vice versa. Insects' behaviors are temperature and humidity-dependent (Holmes, 2010; Ogah et al., 2012; Tochen et al., 2016; Niemelä et al., 2019). This study is confident that automatic synthesis of crickets' songs, temperature, and humidity using methodological (described in Section 2) machine learning approaches can inform farmers of crickets' health (e.g., growth and reproduction rate Ogah et al., 2012), and therefore increase their production.

### 4.4 Training, evaluating and selecting the best classifier

Different machine learning algorithms were trained with single, combined, and spectrogram features. The results are highlighted in Sections 3.4.1, 3.4.2, and 3.4.3. The results from the combined features show that a combination of Chroma+ MFCC had the best performance when trained on RF. With the spectrograms, ResNet152V2 was the best classifier. Generally, image (spectrograms) features had a better performance when trained on deep learning algorithms than numerical features (Chroma, MFCC, LFCC, and their combinations) when trained on shallow machine learning algorithms. This shows that deep learning models perform better than shallow machine learning when voluminous data is available to train the algorithms. Therefore, this study considered ResNet152V2 as the best classifier and was selected for deployment in the decision support system. Since deep learning models do not provide a mechanism for understanding the best variables contributing to the model performance, we considered the best-performing shallow learning classifier (i.e., RF) to further our understanding of feature importance and feature selection.

### 4.5 Feature importance and feature selection

In Section 3.5, it is seen that temperature and humidity had a considerably high significance/contribution to the singing behavior of the crickets. Temperature affects the cricket's ability to express its behavior (including chirping), i.e., low temperature makes insects have few/stagnated behaviors and vice versa. The rate at which the crickets chirp is equally influenced by moisture levels. Higher humidity makes crickets chirp more. Overall, temperature and humidity serve as key environmental factors that shape the physiology, behavior, and ecological interactions of insects. In this research, perhaps the RF machine learning algorithm deciphered the influence of temperature and humidity on the songs produced by the crickets and ranked them as highly significant parameters.

The 12-tonal chroma variations of the cricket songs can be understood from the relationship between the octaves and the frequency. *Gryllus bimaculatus* produces calling songs within a frequency range of 4.7–5.7 kHz (Miyashita et al., 2016; Lankheet et al., 2017), while the courtship song is produced within a frequency range of 15–20 kHz (Miyashita et al., 2016). All these frequencies can be represented in the chroma octave as is in Table 6. Section 3.5 outlined that, C#, and D chroma tonal features were ranked as highly significant by the RF algorithm. Looking at the calling (4,700–5,700 Hz) and courtship (15,000–20,000 Hz) song in the frequencies Table 6, this study notes that they are (close to) equivalent to those of C# and D, and are indeed part of the, target (calling, courtship, and aggression) variables studied. Moreover, C# and D are close in the musical tonal scale, therefore, the cricket song tones are close. Hypothetically the lower-ranked tones, such as A# and B, are far apart in the music tone scale compared to the favorable tones (C# and D) and that could be the reason why they were ranked very low as shown in Table 6. Regarding the MFCC features, this research has not come across any study that explains how the features are organized and gives their respective meanings of the custom column header provided by this research. This study, therefore, was unable to give a scientific explanation behind the model's selection of mfcc11 and mfcc10 variables as highly ranked variables.

**Table 6:** The chromatic octaves 8, 9, and 10 of the 12 tonal variations in Hz can be associated with the cricket songs tonal variations (AudioEngineering, 2023).

**Chroma tone**	**Octave 8**	**Octave 9**	**Octave 10**
C	4,186.01	8,372.02	16,744.04
C#	4,434.92	8,869.84	17,739.69
D	4,698.64	9,397.27	18,794.55
D#	4,978.03	9,956.06	19,912.13
E	5,274.04	10,548.08	
F	5,587.65	11,175.30	
F#	5,919.91	11,839.82	
G	6,271.93	12,543.86	
G#	6,684.88	13,289.75	
A	7,040.00	14,080.00	
A#	7,458.62	14,917.24	
B	7,902.13	15,804.26	

In Section 3.5, the F1-score and accuracy performance metrics of the RF algorithm evaluated with the 6 variables improved from 94.63% and 94.41% to 95.37% respectively, compared to what was illustrated in Table 4. This study notes that RF had consistent results of approximately 95% F1-score while the other algorithms did not. Meaning RF was stable and dependable compared to the others. Moreover, perhaps the less significant features added to the model iteratively, continuously decreased the performance of the RF algorithm. Therefore, 6 features gave the best performance of the model up to 95.63% F1-score and 95.37% accuracy.

### 4.6 Deployment: the decision support system

The pre-trained model discussed in the previous section was integrated at the back end of a web-based application as shown in Section 3.6. The main components, namely the Internet of Things device, the front end, and the back end are discussed below.

#### 4.6.1 Internet of things (IoTs) device

The IoT device implemented in Section 3.6 was built on Raspberry Pi (RaspberryPi, 2023) to capture and record sound, temperature, and humidity using a microphone, temperature, and humidity sensors respectively. Despite the recording of the temperature and humidity every hour during experiments (stated in Section 2.1), in the deployed setup, the timestamp, temperature, humidity, and sound (in the form of a 3 seconds .wav file) were captured after every 5 min. The timestamp, temperature, and humidity were recorded in a CSV file stored in the virtual machine. The name of the .wav file was appended with the timestamp, temperature, and humidity values separated by a # symbol. A wireless fidelity (WIFI) module was integrated into the IoT. Through an internet connection, the files were then transferred to a virtual machine instance in *icipe's* virtual private network using the file transfer protocol (FTP). The IoT was also designed to store data on its local storage in cases where there is no access to the internet but synchronize the data to the cloud servers on the availability of the internet. Each IoT device transferred and updated a specific folder (whose name was the unique identification number of a farmer) in the virtual machine. Logically the folder contained data of a specific farmer and the back and front-end web scripts were tailored to read and process data from those folders based on the unique identification number assigned to the farmer.

#### 4.6.2 Back end

The pre-trained model was saved as a .h5 file and stored in a folder. The data (.wav sound clips) stored in the folders in the cloud servers were linked to each farmer's account. A function extracts the spectrogram features from the 1-second audio file and matches that with the respective temperature and humidity in the CSV metadata file (mentioned in Section 4.6.1). The spectrograms, temperature, and humidity are passed to the pre-trained model (loaded in Django memory) where each set of data goes through the pre-trained model pipeline for necessary preprocessing of the data and prediction. The predicted output is then passed to the front end and displayed on the farmer's dashboard; more details are discussed in the section below.

#### 4.6.3 Front end

The web application (icipe, 2023b) consists of the home page that has a link to the frequently asked questions (FAQ) page and log-in interface. Details of these pages are discussed below:

a) The Home Page: This page contains a narration of the overall function of the system.
b) The FAQ Page: This page is intended to contain information about frequently raised questions about the functioning system and appropriate answers are provided.
c) Log-in Page: At the login interface, the farmer is prompted to provide their username and password and log into the system. The login interface also contains functionality for resetting passwords.
d) Personal Information, Dashboard, and Notification Pages: On successful login, users are taken to the overview page where they can (i) interpret the activities happening in their cricket farm on a dashboard, (ii) go to the account page to edit their personal information, and (iii) go to the notifications page and see the raised alerts; the alerts are similar to what the farmer receives on their registered email in real-time. To interpret the activities happening in the cricket farm in real-time (every 5 min), the front-end system asynchronously passes (by calling an application programming interface micro-service) the farmer's unique identification to the back end for processing and thereafter waits for feedback. The feedback, i.e., the temperature, humidity, and predicted label (calling, calling, or courtship) are displayed on a dashboard. The dashboard's rule-based results of temperature and humidity were interpreted with colors. Blue means low conditions, green means suitable conditions, and red means high conditions. Literature provided different optimal temperature and humidity ranges that crickets prefer. For instance, Busvine (1955), Hanboonsong and Durst (2020), Orinda et al. (2021), and Odhiambo et al. (2022) noted 25–35°C, 26–34°C, 28–32°C, and 28–35°C respectively as optimal temperature ranges. Whereas Hanboonsong and Durst (2020) and Orinda et al. (2021) noted that the optimal humidity range should be between 40–70%RH and 60–75%RH respectively. From this information, this research configured temperature and humidity ranges of 25–35°C and 40–75%RH respectively in the rule-based system.
These findings offer initial insights, prompting farmers to adjust temperature and humidity through measures such as cooling/heating or de/humidifying the rearing area. Conversely, the machine learning insights delve deeper. For instance, negative alerts, such as aggression, prompt the farmer to immediately check the cricket-rearing area, and actions like chasing aggressors can be taken. Positive alerts like calling and courtship indicate good cricket health and growth. With this information, farmers can enhance cricket production as a viable human food source, thereby contributing to alleviating food insecurity.

## 5 Conclusion

An experimental setup was created to collect the humidity, temperature, video, and sound signals of the edible cricket, *Gryllus bimaculatus*. Chroma, Mel Frequency Cepstral Coefficient (MFCC), Linear Frequency Cepstral Coefficients (LFCC), chroma+MFCC, chroma+LFCC, MFCC+LFCC, and spectrograms were extracted from the sound signals. The numerical features were fused with the weather (temperature and humidity) variables and trained on the support vector machine, random forest, and k-nearest neighbors machine learning algorithms. The spectrogram features fused with temperature and humidity were trained on CNN (EfficientNetB4, VGG16, and ResNet152V2) deep-learning pre-trained models. Using machine learning, this study affirms that temperature and humidity highly influence the behavior (chirping) of crickets. Moreover, the frequencies associated with the ranked C# and D chroma features during calling and courtship were also identified. This shows that machine learning was able to identify natural processes associated with insect behavior. Furthermore, we deployed the deep-learning ResNet152V2 pre-trained model at the back end of a web-based decision support system. The system collected data in real-time (every 5 min) and farmers were informed of the predicted (calling, aggression, or courtship) output on the dashboard and notified appropriately. Thereafter, a farmer can put in place appropriate measures (such as cooling, humidifying, getting rid of aggressors, etc.) to avoid the loss of the crickets and improve production. This decision support system can be fine-tuned (by considering more cricket phenomena labels, and wide temperature and humidity ranges) further and adopted by cricket farmers to improve the production of cricket as food for humans and contribute to alleviating food insecurity.

## Data availability statement

The machine learning algorithms scripts are available here (icipe,2023a) and pre-processed data is available here (icipe; https://dmmg.icipe.org/dataportal/dataset/applications-of-deep-learning-algorithms-in-farming-edible-crickets-as-a-source-of-food; 2024-01-01).

## Ethics statement

The studies involving animals were reviewed and approved by the Strathmore University Institutional Scientific and Ethical Review Committee, reference number SU-ISERC1886/23, valid from 6th November 2023 to 5th November 2024 and Research Committee of International Centre of Insect Physiology and Ecology on 22nd of December 2022.

## Author contributions

HK: Data curation, Formal analysis, Investigation, Methodology, Software, Validation, Visualization, Writing – original draft, Writing – review & editing, Conceptualization. HT: Investigation, Writing – original draft, Writing – review & editing. JE: Conceptualization, Data curation, Formal analysis, Funding acquisition, Investigation, Methodology, Resources, Supervision, Validation, Visualization, Writing – original draft, Writing – review & editing. JO: Conceptualization, Data curation, Formal analysis, Funding acquisition, Investigation, Methodology, Resources, Software, Supervision, Validation, Visualization, Writing – original draft, Writing – review & editing. CT: Conceptualization, Formal analysis, Investigation, Methodology, Project administration, Resources, Software, Validation, Visualization, Writing – original draft, Writing – review & editing. KS: Conceptualization, Data curation, Formal analysis, Funding acquisition, Investigation, Methodology, Project administration, Resources, Software, Supervision, Validation, Visualization, Writing – original draft, Writing – review & editing.

## References

[B1] Alexander R. D. (1961). Aggressiveness, territoriality, and sexual behavior in field crickets (Orthoptera: Gryllidae). Behaviour 17, 130–223. 10.1163/156853961X00042

[B2] Ali J. Saleem N. Bourouis S. Alabdulkreem E. Mannai H. E. Dhahbi S. (2024). Spatio-temporal features representation using recurrent capsules for monaural speech enhancement. IEEE Access 12, 21287–21303. 10.1109/ACCESS.2024.3361286

[B3] Alonso J. B. Cabrera J. Shyamnani R. Travieso C. M. Bola nos F. García A.. (2017). Automatic anuran identification using noise removal and audio activity detection. Exp. Syst. Applic. 72, 83–92. 10.1016/j.eswa.2016.12.019

[B4] Amlathe P. (2018). Standard machine learning techniques in audio beehive monitoring: Classification of audio samples with logistic regression, K-nearest neighbor, random forest and support vector machine. PhD thesis, Utah State University.

[B5] Arpitha M. Rani S. K. Lavanya M. (2021). “CNN based framework for classification of mosquitoes based on its wingbeats,” in 2021 Third International Conference on Intelligent Communication Technologies and Virtual Mobile Networks (ICICV) (IEEE), 1–5. 10.1109/ICICV50876.2021.9388600

[B6] Audacity (2023). Free, open source, cross-platform audio software. Available online at: https://www.audacityteam.org/ (accessed May 22, 2023).

[B7] AudioEngineering (2023). Understanding note frequency charts (and why you should be using one). Available online at: https://producelikeapro.com/blog/note-frequency-chart/ (accessed June 5, 2023).

[B8] Breiman L. (2001). Random forests. Mach. Learn. 45, 5–32. 10.1023/A:1010933404324

[B9] Busvine J. (1955). Simple methods for rearing the cricket (*Gryllulus domecstieus L*.) with some observations on speed of development at different temperatures. Proc. R. Entomol. Soc. London 30, 15–18. 10.1111/j.1365-3032.1955.tb00163.x

[B10] Casalicchio G. Molnar C. Bischl B. (2019). “Visualizing the feature importance for black box models,” in Machine Learning and Knowledge Discovery in Databases: European Conference, ECML PKDD 2018, Dublin, Ireland, September 10–14, 2018, Proceedings, Part I 18 (Springer), 655–670. 10.1007/978-3-030-10925-7_40

[B11] Cheney C. R. Larsen M. E. Martinez A. M. Walls D. S. (2018). “What will be the effect of the number of times cricket's chirp if temperature is manipulated in the environment?,” in Undergraduate Research and Creative Activity Symposium (Rhodes College).

[B12] Deng Z. Zhang J. Zhang L. Ye T. Coley Y. Su W. J.. (2022). Fifa: Making fairness more generalizable in classifiers trained on imbalanced data. arXiv preprint arXiv:2206.02792.

[B13] Disha R. A. Waheed S. (2022). Performance analysis of machine learning models for intrusion detection system using gini impurity-based weighted random forest (giwrf) feature selection technique. Cybersecurity 5:1. 10.1186/s42400-021-00103-8

[B14] Django (2023). The web framework for perfectionists with deadlines. Available online at: https://www.djangoproject.com/ (accessed June 5, 2023).

[B15] Doherty J. A. (1985). Temperature coupling and “trade-off”™ phenomena in the acoustic communication system of the cricket, gryllus bimaculatus de geer (gryllidae). J. Exper. Biol. 114, 17–35. 10.1242/jeb.114.1.17

[B16] Dong X. Yan N. Wei Y. (2018). “Insect sound recognition based on convolutional neural network,” in 2018 IEEE 3rd International Conference on Image, Vision and Computing (ICIVC) (IEEE), 855–859. 10.1109/ICIVC.2018.8492871

[B17] Fernández A. Garcia S. Herrera F. Chawla N. V. (2018). Smote for learning from imbalanced data: progress and challenges, marking the 15-year anniversary. J. Artif. Intell. Res. 61, 863–905. 10.1613/jair.1.11192

[B18] Gold B. Morgan N. Ellis D. (2011). Acoustics. John Wiley and Sons, Ltd. 10.1002/9781118142882.part3

[B19] Han J. Pei J. Tong H. (2022). Data Mining: Concepts and Techniques. Cambridge: Morgan Kaufmann.

[B20] Hanboonsong A. Durst P. (2020). Guidance on Sustainable Cricket Farming-A Practical Manual for Farmers and Inspectors. Bangkok: Food &Agriculture Org.

[B21] Holmes L. (2010). Role of abiotic factors on the development and life history of the black soldier fly, Hermetia illucens (l.)(Diptera: Stratiomyidae). Technical report, University of Windsor.

[B22] ICIPE (2023a). An acoustic decision support system for improved farming of crickets. Available online at: https://github.com/icipe-official/Sound-Signals-Analysis-Crickets (accessed June 5, 2023).

[B23] ICIPE (2023b). A decision support system for monitoring behaviour of farmed crickets. Available online at: https://test-dmmg.icipe.org/csa/ (accessed February 1, 2024).

[B24] Jeni L. A. Cohn J. F. De La Torre F. (2013). “Facing imbalanced data-recommendations for the use of performance metrics,” in 2013 Humaine Association Conference on Affective Computing and Intelligent Interaction (IEEE), 245–251. 10.1109/ACII.2013.47 25574450 PMC4285355

[B25] Jones (1966). The acoustic behaviour of the bush cricket pholidoptera griseoaptera: 2 interaction with artificial sound signals. J. Exper. Biol. 45, 31–44. 10.1242/jeb.45.1.31 5969009

[B26] Jonsson T. Montealegre-Z F Soulsbury C. D. Robert D. (2021). Tenors not sopranos: bio-mechanical constraints on calling song frequencies in the mediterranean field-cricket. Front. Ecol. Evolut. 9:647786. 10.3389/fevo.2021.647786

[B27] Joseph V. R. (2022). Optimal ratio for data splitting. Stat. Anal. Data Min. 15, 531–538. 10.1002/sam.11583

[B28] Joseph V. R. Vakayil A. (2022). Split: an optimal method for data splitting. Technometrics 64, 166–176. 10.1080/00401706.2021.1921037

[B29] Kawakita S. Ichikawa K. (2019). Automated classification of bees and hornet using acoustic analysis of their flight sounds. Apidologie 50, 71–79. 10.1007/s13592-018-0619-6

[B30] Kelemu S. Niassy S. Torto B. Fiaboe K. Affognon H. Tonnang H.. (2015). African edible insects for food and feed: inventory, diversity, commonalities and contribution to food security. J. Insects Food Feed 1, 103–119. 10.3920/JIFF2014.001629510743

[B31] Kim J. Oh J. Heo T.-Y. (2021). “Acoustic classification of mosquitoes using convolutional neural networks combined with activity circadian rhythm information,” in International Journal of Interactive Multimedia and Artificial Intelligence. 10.9781/ijimai.2021.08.00934887180

[B32] Kiskin I. Cobb A. D. Wang L. Roberts S. (2020). “Humbug zooniverse: a crowd-sourced acoustic mosquito dataset,” in ICASSP 2020–2020 IEEE International Conference on Acoustics, Speech and Signal Processing (ICASSP) (IEEE), 916–920. 10.1109/ICASSP40776.2020.9053141

[B33] Kramer O. Kramer O. (2013). “K-nearest neighbors,” in Dimensionality Reduction with Unsupervised Nearest Neighbors, ed. O. Kramer (Berlin: Springer), 13–23. 10.1007/978-3-642-38652-7_2

[B34] Kubernetes (2023). An open-source system for automating deployment, scaling, and management of containerized applications. Available online at: https://kubernetes.io/ (accessed June 5, 2023).

[B35] Lange K. W. Nakamura Y. (2021). Edible insects as future food: chances and challenges. J. Future Foods 1, 38–46. 10.1016/j.jfutfo.2021.10.00134977112

[B36] Lankheet M. Cerkvenik U. Larsen O. Van Leeuwen J. L. (2017). Frequency tuning and directional sensitivity of tympanal vibrations in the field cricket gryllus bimaculatus. J. R. Soc. Interf. 14:20170035. 10.1098/rsif.2017.0035 28298611 PMC5378147

[B37] Le-Qing Z. (2011). “Insect sound recognition based on MFCC and PNN,” in 2011 International Conference on Multimedia and Signal Processing. 10.1109/CMSP.2011.100

[B38] Lin C.-C. Hedwig B. (2021). Wing movements underlying sound production in calling, rivalry, and courtship songs of the cricket gryllus bimaculatus (degeer). J. Insect Physiol. 134:104299. 10.1016/j.jinsphys.2021.104299 34418404

[B39] Liu Y. Pu H. Sun D.-W. (2021). Efficient extraction of deep image features using convolutional neural network (CNN) for applications in detecting and analysing complex food matrices. Trends Food Sci. Technol. 113, 193–204. 10.1016/j.tifs.2021.04.04237754954

[B40] Magara H. J. Niassy S. Ayieko M. A. Mukundamago M. Egonyu J. P. Tanga C. M.. (2021). Edible crickets (Orthoptera) around the world: distribution, nutritional value, and other benefits–a review. Front. Nutr. 7:257. 10.3389/fnut.2020.537915 33511150 PMC7835793

[B41] McFee B. McVicar M. Faronbi D. Roman I. Gover M. Balke S.. (2023). Librosa. Zenodo. 10.5281/zenodo.7746972

[B42] Mhatre N. Balakrishnan R. (2006). Male spacing behaviour and acoustic interactions in a field cricket: implications for female mate choice. J. Animal Behav. 72, 1045–1058. 10.1016/j.anbehav.2006.02.022

[B43] Miyashita A. Kizaki H. Sekimizu K. Kaito C. (2016). No effect of body size on the frequency of calling and courtship song in the two-spotted cricket, *Gryllus bimaculatus*. PLoS ONE 11:e0146999. 10.1371/journal.pone.0146999 26785351 PMC4718538

[B44] Niemelä P. T. Niehoff P. P. Gasparini C. Dingemanse N. J. Tuni C. (2019). Crickets become behaviourally more stable when raised under higher temperatures. Behav. Ecol. Sociobiol. 73, 1–12. 10.1007/s00265-019-2689-5

[B45] Noda J. J. Travieso C. M. Sánchez-Rodríguez D. Dutta M. K. Singh A. (2016). “Using bioacoustic signals and support vector machine for automatic classification of insects,” in 2016 3rd International Conference on Signal Processing and Integrated Networks (SPIN) (IEEE), 656–659. 10.1109/SPIN.2016.7566778

[B46] Noda J. J. Travieso-González C. M. Sánchez-Rodríguez D. Alonso-Hernández J. B. (2019). Acoustic classification of singing insects based on MFCC/LFCC fusion. Appl. Sci. 9:4097. 10.3390/app9194097

[B47] Odhiambo M. Ochia C. Okuto E. (2022). Effects of temperature on the development and survival of cricket species; *Acheta domesticus* and *Gryllus bimaculatus* (orthoptera: Gryllidae). East Afr. J. Agric. Biotechnol. 5, 176–189. 10.37284/eajab.5.1.834

[B48] Ogah E. Owoh E. Nwilene F. Ogbodo E. (2012). Effect of abiotic factors on the incidence of african rice gall midge, orseolia oryzivora and its parasitism by platygaster diplosisae and aprostocetus procerae. J. Biol. Agric. Healthcare 2, 60–65.

[B49] Orinda M. A. Oloo J. Magara H. J. Ayieko M. (2021). Cricket Rearing Handbook. London: Society for Science and Education (United Kingdom). 10.14738/eb.86.2020

[B50] Pedregosa F. Varoquaux G. Gramfort A. Michel V. Thirion B. Grisel O.. (2011). Scikit-learn: machine learning in Python. J. Mach. Learn. Res. 12, 2825–2830. 10.48550/arXiv.1201.0490

[B51] Phung Q. V. Ahmad I. Habibi D. Hinckley S. (2017). Automated insect detection using acoustic features based on sound generated from insect activities. Acoust. Austr. 45, 445–451. 10.1007/s40857-017-0095-6

[B52] Potamitis I. Eliopoulos P. Rigakis I. (2017). Automated remote insect surveillance at a global scale and the internet of things. Robotics 6:19. 10.3390/robotics6030019

[B53] Potamitis I. Ganchev T. Fakotakis N. (2007). “Automatic acoustic identification of crickets and cicadas,” in 2007 9th International Symposium on Signal Processing and Its Applications (IEEE), 1–4. 10.1109/ISSPA.2007.4555462

[B54] RaspberryPi (2023). Raspberrypi. Available online at: https://www.raspberrypi.com/ (accessed June 5, 2023).

[B55] Silva D. F. De Souza V. M. Batista G. E. Keogh E. Ellis D. P. (2013). “Applying machine learning and audio analysis techniques to insect recognition in intelligent traps,” in 2013 12th International Conference on Machine Learning and Applications (IEEE), 99–104. 10.1109/ICMLA.2013.24

[B56] Srygley R. B. (2014). Effects of temperature and moisture on mormon cricket reproduction with implications for responses to climate change. J. Insect Physiol. 65, 57–62. 10.1016/j.jinsphys.2014.05.005 24831180

[B57] Steiniger Y. Stoppe J. Meisen T. Kraus D. (2020). “Dealing with highly unbalanced sidescan sonar image datasets for deep learning classification tasks,” in Global Oceans 2020: Singapore-US Gulf Coast (IEEE), 1–7. 10.1109/IEEECONF38699.2020.9389373

[B58] Suthaharan S. Suthaharan S. (2016). “Support vector machine,” in Machine Learning Models and Algorithms for Big Data Classification: Thinking With Examples for Effective Learning, ed. S. Suthaharan (New York, NY: Springer), 207–235. 10.1007/978-1-4899-7641-3_9

[B59] Tanga C. Egonyu J. Beesigamukama D. Niassy S. Emily K. Magara H. J.. (2021). Edible insect farming as an emerging and profitable enterprise in east africa. Curr. Opin. Insect Sci. 48, 64–71. 10.1016/j.cois.2021.09.007 34649017

[B60] Tey W. T. Connie T. Choo K. Y. Goh M. K. O. (2022). Cicada species recognition based on acoustic signals. Algorithms 15:358. 10.3390/a15100358

[B61] Tochen S. Woltz J. M. Dalton D. Lee J. Wiman N. Walton V. (2016). Humidity affects populations of *Drosophila suzukii* (Diptera: Drosophilidae) in blueberry. J. Appl. Entomol. 140, 47–57. 10.1111/jen.1224731207075

[B62] Ulagaraj S. (1976). Sound production in mole crickets (orthoptera: Gryllotalpidae: Scapteriscus). Ann. Entomol. Soc. Am. 69, 299–306. 10.1093/aesa/69.2.29915802674

[B63] van Huis A. (2013). Potential of insects as food and feed in assuring food security. Ann. Rev. Entomol. 58, 563–583. 10.1146/annurev-ento-120811-153704 23020616

[B64] Verner D. Roos N. Halloran A. M. S. Surabian G. M. Tebaldi E. Ashwill M. S.. (2021). Insect and Hydroponic Farming in Africa: The New Circular Food Economy (Vol. 2): Overview (French). Washington, DC: World Bank Group. 10.1596/978-1-4648-1766-3

[B65] Vujović Ž. (2021). Classification model evaluation metrics. Int. J. Adv. Comput. Sci. Applic. 12, 599–606. 10.14569/IJACSA.2021.0120670

[B66] Wang S. Liu W. Wu J. Cao L. Meng Q. Kennedy P. J. (2016). “Training deep neural networks on imbalanced data sets,” in 2016 International Joint Conference on Neural Networks (IJCNN) (IEEE), 4368–4374. 10.1109/IJCNN.2016.7727770

[B67] Yazgaç B. G. Kırcı M. Kıvan M. (2016). “Detection of sunn pests using sound signal processing methods,” in 2016 Fifth International Conference on Agro-Geoinformatics (Agro-Geoinformatics) (IEEE), 1–6. 10.1109/Agro-Geoinformatics.2016.7577694

[B68] Zamanian H. Pourghassem H. (2017). “Insect identification based on bioacoustic signal using spectral and temporal features,” in 2017 Iranian Conference on Electrical Engineering (ICEE) (IEEE), 1785–1790. 10.1109/IranianCEE.2017.7985340

[B69] Zhang M. Yan L. Luo G. Li G. Liu W. Zhang L. (2021). “A novel insect sound recognition algorithm based on MFCC and CNN,” in 2021 6th International Conference on Communication, Image and Signal Processing (CCISP) (IEEE), 289–294. 10.1109/CCISP52774.2021.9639350

[B70] Zhang N. Guo M. (2010). “Recognition of fruit fly wings vibration sound based on HMM,” in 2010 2nd International Conference on Information Engineering and Computer Science (IEEE), 1–4. 10.1109/ICIECS.2010.5678369

[B71] Zulko (2023). Video, Editing, Audio, Compositing, Ffmpeg. Available online at: https://pypi.org/project/moviepy/ (accessed May 22, 2023).

